# A Telerehabilitation-Based Fine Motor Training Program for Children With Inattentive Attention-Deficit/Hyperactivity Disorder: Randomized Controlled Trial

**DOI:** 10.2196/97365

**Published:** 2026-07-23

**Authors:** Feilong Zhu, Yue Sun, Dongqing Kuang, Xiaotong Zhu, Xiaoyu Bi, Yufeng Wang, Li Yang, Yuanchun Ren, Ming Zhang

**Affiliations:** 1 School of Sports Medicine and Rehabilitation, Beijing Sport University Beijing China; 2 Department of Rehabilitation, Guang’anmen Hospital, China Academy of Chinese Medical Sciences Beijing China; 3 College of Physical Education and Sports, Beijing Normal University Beijing China; 4 School of Physical Education Science, Tianjin Normal University Tianjin China; 5 School of Health Care Technology, Dalian Neusoft University of Information Dalian China; 6 NHC Key Laboratory of Mental Health (Peking University), National Clinical Research Center for Mental Disorders, Peking University Sixth Hospital Beijing China; 7 The Affiliated Xuzhou Rehabilitation Hospital of Xuzhou Medical University Xuzhou China

**Keywords:** ADHD, attention-deficit/hyperactivity disorder, executive functions, fine motor training, inattention symptoms, randomized controlled trial, RCT

## Abstract

**Background:**

Children with inattentive attention-deficit/hyperactivity disorder (ADHD) often present with impairments in executive functions and fine motor skills in addition to core inattentive symptoms. However, evidence remains limited regarding structured telerehabilitation-based fine motor training for these outcomes.

**Objective:**

This study aims to examine the effects of a 12-week telerehabilitation-based fine motor training program on inattention symptoms, executive functions, and fine motor skills in children with inattentive ADHD.

**Methods:**

This randomized controlled trial included 66 children aged 6 to 10 years with inattentive ADHD. Participants were randomly assigned to a telerehabilitation-based fine motor training group (n=33) or a waitlist control group (n=33). The intervention was delivered live online 3 times per week, 60 minutes per session, for 12 weeks. Assessments were conducted at baseline, immediately after the intervention, and at the 3-month follow-up. Outcomes included parent-reported inattention symptoms, executive functions, and fine motor skills. Linear mixed-effects models were used for the primary analysis, and an exploratory mediation analysis was conducted using postintervention values at 12 weeks.

**Results:**

Compared with the waitlist control group, the intervention group showed greater reductions in parent-reported inattention symptoms at 12 weeks (mean difference [MD]=−3.85, 95% CI −5.01 to −2.68; Hedges *g*=−1.13; *P*<.001) and 24 weeks (MD=−2.00, 95% CI −3.17 to −0.83; Hedges *g*=−0.59; *P*<.001). For executive functions, between-group differences favored the intervention group for inhibitory control at 12 weeks (MD=−11.62, 95% CI −19.78 to −3.46; Hedges *g*=−0.83; *P*=.006) and 24 weeks (MD=−8.88, 95% CI −17.04 to −0.72; Hedges *g*=−0.63; *P*=.03), immediate memory at both time points, and cognitive flexibility at both time points. Delayed memory showed a significant between-group difference only at 12 weeks (MD=3.03, 95% CI 0.57 to 5.49; Hedges *g*=0.65; *P*=.02). For fine motor skills, between-group differences favored the intervention group for manual dexterity at 12 weeks (MD=2.97, 95% CI 1.12 to 4.73; Hedges *g*=1.17; *P*=.001) and 24 weeks (MD=2.94, 95% CI 1.18 to 4.70; Hedges *g*=1.16; *P*=.001), and for hand-eye coordination at 12 weeks (MD=2.48, 95% CI 0.68 to 4.29; Hedges *g*=1.06; *P*=.007) and 24 weeks (MD=2.09, 95% CI 0.28 to 3.90; Hedges *g*=0.89; *P*=.02). Writing skills improved at 12 weeks but not at follow-up. An exploratory mediation analysis using week-12 postintervention values suggested a statistical indirect association through inhibitory control (*β*=−0.85, 95% CI −1.85 to −0.08; *P*=.05). However, post hoc lagged exploratory mediation analyses did not support longitudinal indirect effects through inhibitory control or other executive function outcomes.

**Conclusions:**

A 12-week telerehabilitation-based fine motor training program may be a feasible and potentially beneficial adjunctive intervention for children with inattentive ADHD. The findings suggest potential benefits for parent-reported inattention symptoms, selected executive function outcomes, and fine motor skills but should be interpreted cautiously given the waitlist control design, parent-reported symptom outcome, and exploratory mediation analysis.

**Trial Registration:**

Chinese Clinical Trial Registry ChiCTR2200065413; https://www.chictr.org.cn/showproj.html?proj=182412

## Introduction

Attention-deficit/hyperactivity disorder (ADHD) is one of the most common neurodevelopmental disorders in childhood, with an estimated worldwide prevalence of approximately 5%-7% [1-3]. It is characterized by developmentally inappropriate symptoms of inattention, hyperactivity, and impulsivity. Beyond these core behavioral symptoms, children with ADHD frequently present with impairments in executive functions and motor performance, which are associated with difficulties in academic achievement, daily functioning, and social adaptation [4-6]. Executive dysfunction, particularly in inhibitory control, working memory, and cognitive flexibility, is widely regarded as an important feature of ADHD and a relevant target for intervention [7,8]. The inattentive presentation of ADHD deserves particular attention because it is closely related to difficulties in sustained attention, task persistence, response monitoring, and goal-directed regulation [1]. These difficulties are often accompanied by weaknesses in executive control, which may contribute to symptom expression and functional difficulties in daily life [9].

Motor difficulties are also common in children with ADHD. Previous studies have shown that children with ADHD often perform more poorly than typically developing peers on measures of motor coordination, balance, manual dexterity, and fine motor skills [6,10-15]. Fine motor deficits may be particularly relevant for children with predominantly inattentive symptoms because many everyday tasks require distal hand control, visual-motor coordination, sustained precision, and attention to task demands [5]. These motor problems are clinically meaningful because they may interfere with handwriting, classroom activities, tool use, self-care routines, and other daily functional activities [5]. Moreover, executive function difficulties have been linked to both academic writing skills and activities of daily living in children with ADHD, suggesting that cognitive and motor difficulties may jointly shape children’s real-life functioning [8,16,17].

From an occupational performance perspective, fine motor difficulties should not be viewed only as isolated impairments in hand function. Rather, they may restrict children’s participation in meaningful everyday occupations, including schoolwork, play, self-care, and home routines [18,19]. Activities such as handwriting, drawing, cutting, manipulating small objects, using utensils, organizing learning materials, fastening buttons, and completing classroom tasks require coordinated fine motor control together with attention, inhibition, sequencing, and performance monitoring. Therefore, interventions targeting fine motor skills may have broader translational relevance when they are embedded in meaningful activity contexts and interpreted in relation to children’s functional engagement. This perspective is consistent with occupation-centered pediatric rehabilitation approaches, which emphasize activity performance, goal-directed practice, and transfer of learned skills to daily contexts [18,19].

Exercise-based and movement-based interventions are increasingly recognized as promising nonpharmacological approaches for children with ADHD. Previous randomized controlled trials (RCTs) and reviews have shown that aerobic exercise and cognitive-motor programs can improve ADHD symptoms and executive functions, and some studies have suggested that executive functions may mediate symptom changes [8,20,21]. However, less is known about interventions that specifically target fine motor and graphomotor difficulties, despite the relevance of these difficulties to school- and home-based activities in children with ADHD. Fine motor training may represent a complementary rehabilitation approach because it directly engages hand control, visual-motor integration, sequencing, response inhibition, speed-accuracy regulation, and performance monitoring. These processes overlap with executive function demands and may be relevant for children with inattentive ADHD [22]. Because fine motor activities require limited space and simple materials, they may also be suitable for home-based telerehabilitation delivery. Importantly, this rationale does not imply that fine motor training is superior to broader exercise-based or cognitive-motor interventions; rather, it highlights a distinct and clinically relevant intervention target that requires further investigation.

Telerehabilitation may provide a feasible way to deliver fine motor training in ecologically relevant settings. Compared with conventional center-based rehabilitation, home-based online delivery can reduce barriers related to travel, scheduling, cost, and access to trained providers [7]. It may also allow children to practice skills in the same environment in which many daily routines occur. Fine motor activities are particularly suitable for remote delivery because they generally require limited space and simple household materials. By incorporating activities such as object sorting, folding, cutting, tracing, writing-related practice, and home-based manipulation tasks, a telerehabilitation-based fine motor program may provide repeated opportunities to practice motor and executive processes in contexts closer to children’s everyday occupations [13,14,23]. However, evidence remains limited regarding structured, therapist-guided remote fine motor interventions for children with inattentive ADHD, particularly studies that include follow-up assessment.

Therefore, this RCT investigated the effects of a 12-week telerehabilitation-based fine motor training program in children aged 6 to 10 years with inattentive ADHD. Compared with a waitlist control group, we examined whether the intervention was associated with greater improvements in inattention symptoms, executive functions, and fine motor skills immediately after treatment and at the 3-month follow-up. We also conducted exploratory statistical mediation analyses to examine whether executive function outcomes showed statistical indirect associations between intervention allocation and inattention symptoms.

## Methods

### Ethical Considerations

The study was approved by the Ethics Committee of Beijing Normal University (approval number 2107) and Xuzhou Rehabilitation Hospital (approval number XK-LW-20221020-002), conducted in accordance with the Declaration of Helsinki, and registered with the Chinese Clinical Trial Registry (ChiCTR2200065413). Written informed consent was obtained from the parents or legal guardians of all participants before enrollment. Data analysis and reporting followed the CONSORT (Consolidated Standards of Reporting Trials) guidelines [24]. The CONSORT-EHEALTH (Consolidated Standards of Reporting Trials of Electronic and Mobile Health Applications and Online Telehealth) submission/publication form is available in Multimedia Appendix 1. To protect participants’ privacy and confidentiality, each participant was assigned a unique study identification number, and personally identifiable information was stored separately from the research data. Electronic data were stored in password-protected files and were accessible only to authorized members of the research team. All data used for statistical analysis and reporting were deidentified, and no information that could identify individual participants was included in any publication or presentation. Participants did not receive any financial or material compensation for taking part in the study.

### Study Design

This was a 2-arm, parallel-group, assessor-blinded RCT conducted between March 2024 and March 2026. Eligible children were randomly assigned in a 1:1 ratio to either a telerehabilitation-based fine motor training group or a waitlist control group. Assessments were conducted at baseline, immediately after the 12-week intervention, and at the 3-month follow-up.

### Participants and Recruitment

Participants were recruited online through study posters disseminated by the research team via social media platforms and parent WeChat groups for children with ADHD. Interested families contacted the research team and underwent an initial eligibility screening. All enrolled children had previously received a hospital-based diagnosis of inattentive ADHD before entering the study. The recruitment age range was limited to 6 to 10 years for developmental and practical reasons. Children in this age range are typically in primary school and are expected to perform increasingly complex fine motor and graphomotor tasks, such as handwriting, drawing, cutting, tool use, and organizing learning materials. Executive functions, including inhibition, working memory, and cognitive flexibility, also undergo substantial development during this period. Therefore, this age range was considered appropriate for evaluating whether structured fine motor training could influence both motor and executive outcomes in children with inattentive ADHD.

Children were eligible for inclusion if they met all of the following criteria: (1) were aged 6 to 10 years, (2) had a prior clinical diagnosis of inattentive ADHD made in a hospital setting, (3) had an IQ of at least 80 as assessed by the Wechsler Intelligence Scale [25], (4) were able to understand task instructions and complete the assessments and online training, and (5) had parental support to participate in the home-based online sessions and follow-up assessments.

Children were excluded if they had comorbid autism spectrum disorder, epilepsy, intellectual disability, major sensory or motor impairment, or any other neurological, psychiatric, or physical condition that could interfere with safe participation or valid assessment. Children receiving stimulant medication were permitted to participate, provided that their medication status was documented. To minimize the acute effects of stimulant medication on neuropsychological performance, participants taking stimulant medication were required to discontinue the medication for at least 48 hours before assessment.

### Sample Size Calculation

Because no directly comparable randomized trials of telerehabilitation-based fine motor training in children with inattentive ADHD were available, the sample size was estimated using the best available evidence from previous exercise intervention studies involving children with ADHD. Specifically, a previously reported standardized mean difference (MD) of 0.68 for improvements in ADHD-related symptoms and executive functions following exercise interventions was used as the expected effect size [20]. Although the primary analysis was conducted using linear mixed-effects models to account for repeated measurements and within-participant correlations, the 2-tailed independent-samples *t* test was used as a pragmatic approximation for estimating the expected between-group difference. The calculation was performed using G*Power software (version 3.1.9.7) based on an independent-samples *t* test, with a 2-sided α of .05 and statistical power of 80%. The minimum required sample size was estimated to be 56 participants. Assuming an attrition rate of 15%, the target enrollment was set at 66 participants. The sample size calculation was based primarily on the expected intervention effect on ADHD-related symptoms and executive functions. The study was not specifically powered for secondary outcomes, mediation analysis, or subgroup comparisons; therefore, these analyses were considered exploratory.

### Randomization, Allocation Concealment, and Blinding

After baseline assessment, participants were randomly assigned in a 1:1 ratio to either the intervention group or the waitlist control group. The randomization sequence was generated using R software (version 4.4.0; R Foundation for Statistical Computing) by a researcher who was not involved in recruitment, intervention delivery, or outcome assessment. Allocation concealment was maintained using sequentially numbered, opaque, sealed envelopes. Each envelope was opened only after eligibility had been confirmed and baseline assessment had been completed. Because of the behavioral nature of the intervention, participants, caregivers, and instructors could not be blinded to group allocation. However, outcome assessors were not involved in intervention delivery and remained blinded to group assignment throughout the trial. Assessment appointments were scheduled separately from intervention sessions, and assessors did not have access to the randomization list or intervention attendance records. Families were instructed not to disclose their allocation during assessments.

### Intervention and Control Conditions

Medication status and daily moderate-to-vigorous physical activity (MVPA) were recorded at baseline. Participants were asked to maintain their usual medication and physical activity patterns during the study period and not to initiate any additional rehabilitation, occupational therapy, or structured fine motor training outside the trial.

### Telerehabilitation-Based Fine Motor Training

The intervention consisted of a structured telerehabilitation-based fine motor training program delivered live via Tencent Meeting. Sessions were conducted 3 times per week, 60 minutes per session, for 12 consecutive weeks, resulting in 36 scheduled sessions. The program was delivered in small online groups by trained instructors. Parents were asked to provide supervision when needed, particularly for camera positioning, preparation of materials, and ensuring safety in the home environment.

Each session followed a standardized intervention manual. The program aimed to improve fine motor performance while simultaneously engaging attentional control and executive processes relevant to inattentive ADHD. The training content included 4 components: hand dexterity training, hand-eye coordination training, integrated fine motor-cognitive training, and writing skills training. These components were included throughout the intervention period, with task difficulty progressively adjusted according to each child’s performance and tolerance.

The hand dexterity component focused on finger control, bilateral hand coordination, movement precision, and manipulation speed. Representative activities included finger opposition sequences, isolated finger tapping, thumb-index pinching, bead or coin transfer, clipping tasks, peg placement, buttoning and unbuttoning, folding paper, cutting or tearing along lines, and object sorting using tweezers or chopsticks. Progression was achieved by increasing task speed, reducing object size, extending sequence length, or adding bimanual coordination demands.

The hand-eye coordination component emphasized visuomotor integration, spatial accuracy, visual tracking, and timed upper-limb responses. Activities included tracing visual paths, dot-to-dot connections, target pointing, object placement into marked areas, cup stacking, ring placement, guided object transfer, and ball-based tasks such as throwing and catching a tennis ball. These activities were selected to strengthen the coordination between visual input and manual output, which is relevant to school-based tasks and daily functioning.

The integrated fine motor-cognitive component embedded executive demands within fine motor tasks. For inhibitory control, children were asked to start or stop movements according to specific signals, suppress responses to distractor cues, or perform go/no-go-style hand actions. For working memory, children completed multistep hand sequences, reproduced patterns after a short delay, or manipulated objects according to instructions that had to be remembered and updated. For cognitive flexibility, tasks required switching response rules, changing sorting criteria, alternating hand use, or adapting movement sequences when new instructions were introduced.

The writing skills component focused on graphomotor control and functional handwriting-related abilities. Activities included pencil grasp practice, line and shape tracing, stroke direction training, copying simple geometric figures, connecting broken lines, visual-spatial alignment exercises, and age-appropriate writing drills emphasizing neatness, pressure control, spacing, and movement fluency. Early sessions focused on posture, grip, and basic control, whereas later sessions incorporated more complex copying and timed written output.

Although the intervention included structured fine motor exercises, the tasks were selected with consideration of their relevance to children’s everyday occupational performance. Activities such as buttoning, folding, cutting, using chopsticks or tweezers, organizing stationery, tracing, copying figures, and writing-related drills reflected functional demands encountered in self-care, schoolwork, play, and home routines. Thus, the intervention was designed not only to train isolated hand function but also to provide repeated practice of fine motor control in activity contexts relevant to children’s daily participation.

A typical 60-minute session began with a 5- to 10-minute warm-up involving simple finger exercises, followed by the main training blocks and brief transitions between components. Instructors provided live demonstrations, real-time correction, encouragement, pacing adjustment, and task progression throughout the session. Homework was assigned after each session to extend practice beyond the live online class and support transfer to daily activities. Homework usually involved repeating selected session activities or completing fine motor tasks embedded in home routines, such as sorting small household objects, folding clothes, using utensils, organizing stationery, or participating in simple household chores requiring hand manipulation. Parents were asked to upload photos or short videos in the study chat group to document homework completion and provide feedback.

### Intervention Fidelity and Adherence

Several procedures were used to support intervention fidelity. All instructors received training before the trial and delivered the program according to the standardized manual. Each session followed a structured plan specifying the training components, task sequence, approximate duration, progression criteria, and safety considerations. During live online sessions, instructors observed children’s performance through the video interface, provided real-time correction, and adjusted task difficulty according to each child’s performance and tolerance. The research team held regular meetings to review implementation issues and maintain consistency across instructors.

Attendance was recorded for every scheduled online session. For descriptive purposes, intervention adherence was defined a priori as attendance at ≥80% of scheduled sessions, corresponding to participation in at least 29 of the 36 sessions. Homework completion was monitored through parent-uploaded photos or short videos. These procedures were used to support adherence and delivery quality in the online setting.

### Waitlist Control Group

Children in the waitlist control group did not receive the fine motor training during the 12-week intervention period and continued their usual routines. They completed the same assessment schedule as the intervention group at baseline, immediately after the intervention, and 3-month follow-up. For ethical reasons, families in the waitlist control group were offered access to the intervention materials after completion of the study.

### Outcome Measures

Assessments were conducted at 3 time points: baseline, immediately after the 12-week intervention, and 3 months after the end of the intervention. Demographic and clinical information collected at baseline included age, sex, height, weight, BMI, IQ, medication status, and daily MVPA. The outcome measures included inattention symptom severity, executive functions, and fine motor skills.

Daily MVPA was objectively measured using a triaxial accelerometer (ActiGraph GT9X). Accelerometer data were sampled at 30 Hz and aggregated into 15-second epochs using ActiLife software. MVPA was defined using age-specific Evenson cut points, with ≥2296 counts per minute classified as MVPA. Daily MVPA was calculated as the average number of minutes per day spent in MVPA across valid monitoring days and was included as a baseline covariate in the linear mixed-effects models because habitual physical activity may influence executive function and motor outcomes.

### Inattention Symptoms

Inattention symptoms were assessed using the inattention subscale of the Swanson, Nolan, and Pelham IV Rating Scale (SNAP-IV), completed by parents. The SNAP-IV is a widely used parent-report instrument for evaluating ADHD symptom severity and treatment response, and the Chinese version has shown acceptable reliability and validity [26]. Higher scores indicate greater symptom severity. Because the SNAP-IV was completed by parents, this measure was considered a parent-reported symptom outcome rather than an objective measure of attention.

### Executive Functions

Executive functions were assessed across 3 domains: inhibitory control, working memory–related visual memory performance, and cognitive flexibility.

Inhibitory control was assessed using a paper-based Stroop Color and Word Test [27]. The test was administered individually by trained assessors in a quiet testing room according to standardized procedures. Before formal testing, children received standardized instructions and completed practice trials to ensure that they understood the task requirements. The task material consisted of Chinese color words printed in incongruent ink colors. Children were instructed to name the ink color of each word as quickly and accurately as possible while suppressing the automatic tendency to read the word itself. This color-word interference condition was used to assess inhibitory control. If an error occurred, the child was asked to correct it before continuing, and the total completion time was recorded. Longer completion times indicated poorer inhibitory control. The task materials used in this study are available in Multimedia Appendix 2.

Working memory–related visual memory performance was assessed using the Rey-Osterrieth Complex Figure Test, administered with the study-specific materials provided in Multimedia Appendix 2 [28]. The test was administered individually using standardized test materials and instructions. Children were first shown Rey’s complex figure for 30 seconds and instructed to observe it carefully. They were then asked to reproduce the figure from memory immediately after presentation. After a 30-minute interval, during which no additional exposure to the figure was provided, children were asked to reproduce the figure again from memory. The reproduced figures were scored according to standardized criteria based on the accuracy, placement, and structural organization of the figure elements. The immediate detail score and delayed detail score, each ranging from 0 to 36, were used as the outcome measures and are referred to as immediate memory and delayed memory in this study. Higher scores indicated better visual memory performance.

Cognitive flexibility was assessed using a paper-based Trail Making Test administered with the study-specific materials provided in Multimedia Appendix 2 [29]. The test was administered individually in a quiet testing room according to standardized study procedures. Standardized instructions and practice items were provided before formal testing. The task consisted of 25 circles, including numbers from 1 to 13 and letters from A to L. Children were instructed to connect the circles by alternating between numbers and letters in sequence as quickly and accurately as possible, for example, 1-A-2-B-3-C. If an error occurred, the assessor immediately pointed it out and instructed the child to return to the correct sequence before continuing. Completion time was used as the primary indicator of cognitive flexibility, with shorter completion times indicating better cognitive flexibility.

### Fine Motor Skills

Fine motor skills were assessed across 3 domains: manual dexterity, hand-eye coordination, and writing skill.

Manual dexterity and hand-eye coordination were assessed using the Movement Assessment Battery for Children–Second Edition [30]. This is a standardized instrument widely used to evaluate motor performance in children and has been applied extensively in both clinical and research settings. In this study, the manual dexterity and aiming and catching components were used to reflect hand dexterity and hand-eye coordination, respectively. Higher scores indicated better fine motor performance.

Writing skills were assessed using the Tseng Handwriting Problem Checklist (THPC) [31]. This is a handwriting assessment tool developed for school-aged children and is used to evaluate handwriting-related difficulties across 24 items. It is considered an effective instrument for identifying 6 dimensions of handwriting problems, including construction, sequencing, behavior, accuracy, movement, and directionality. In this study, the total score was used to reflect overall writing ability, with lower scores indicating better writing performance.

### Adverse Events

Adverse events were monitored throughout the intervention period. Parents were encouraged to report any physical discomfort, fatigue, emotional distress, or other unexpected events occurring during or after training sessions. All reported events were documented and reviewed by the study team.

### Statistical Analysis

All statistical analyses were conducted using R software (version 4.4.0). Statistical significance was set at a 2-sided *P*<.05. Continuous variables are presented as mean (SD), and categorical variables are summarized as number (%). Baseline demographic and clinical characteristics were summarized by group to assess the comparability of the randomized sample.

The primary analysis followed the intention-to-treat principle, with all randomized participants analyzed in their originally assigned groups. Repeated outcome data collected at baseline, immediately after the 12-week intervention, and at the 3-month follow-up were analyzed using linear mixed-effects models. Missing outcome data were handled within the mixed-effects model framework using maximum likelihood estimation, which allowed all available repeated-measures data to be included under the missing-at-random assumption. In each model, group, time, and the group-by-time interaction were included as fixed effects, and participant ID was included as a random effect to account for within-participant correlations across repeated assessments. Age, sex, IQ, medication status, and daily MVPA were included as covariates. The main effects of group and time and the group-by-time interaction were reported for each outcome. The group-by-time interaction was used to evaluate whether changes over time differed between the intervention and waitlist control groups. Post hoc pairwise comparisons were performed using estimated marginal means. To account for multiple post hoc comparisons, *P* values from pairwise comparisons were adjusted using the false discovery rate method. Standardized between-group effect sizes were calculated as Hedges *g* based on change scores from baseline to each follow-up time point. Effect sizes were interpreted as trivial (<0.20), small (0.20-0.49), moderate (0.50-0.79), or large (≥0.80).

Clinical response was defined as a reduction of at least 20% from baseline in the SNAP-IV inattention score, and marked response was defined as a reduction of at least 40% [32]. To examine the robustness of the main findings, a sensitivity analysis was performed after excluding children who were receiving ADHD medication during the trial. In addition, an adherence-based sensitivity analysis was conducted to examine the potential influence of incomplete adherence. Participants in the intervention group who attended fewer than 80% of the scheduled sessions were excluded from this analysis.

Prespecified subgroup analyses were conducted by age group, with participants divided into younger children aged 6 to 8 years and older children aged 9 to 10 years. This grouping was selected to broadly distinguish early primary school–aged children from older primary school–aged children, given developmental changes in fine motor control, handwriting demands, classroom participation, and executive functions during this period. However, these subgroup analyses were exploratory. The study was not powered to detect age-specific treatment effects or formal age-by-treatment interaction effects, and subgroup estimates were expected to have limited precision. Sex-stratified analyses were not performed because of the marked sex imbalance and the limited number of girls, which would have resulted in insufficient statistical power for reliable subgroup comparisons.

To explore possible explanatory pathways linking the intervention to inattention symptoms, correlation and exploratory statistical mediation analyses were conducted using postintervention values at week 12. Pearson correlation coefficients were first calculated to examine the associations between executive function outcomes and inattention scores at week 12. Simple mediation models were then fitted with treatment allocation coded as a binary independent variable (0=waitlist control group; 1=intervention group), postintervention executive function outcomes entered as candidate mediators, and the postintervention inattention score entered as the dependent variable. Candidate mediators included inhibitory control, immediate memory, delayed memory, and cognitive flexibility. Total, direct, and indirect effects were estimated and reported as β coefficients with corresponding 95% CIs. Because the candidate mediators and inattention symptoms were assessed at the same postintervention time point, these analyses were considered exploratory and were interpreted as statistical mediation rather than evidence of causal mediation. To partially address the limitation of temporal ordering, we further conducted post hoc lagged exploratory mediation analyses in the intention-to-treat sample. In these analyses, each week-12 executive function outcome was entered as a candidate mediator, and week-24 inattention symptoms were entered as the subsequent outcome, with adjustment for the corresponding baseline executive function value and baseline inattention symptoms. A change-score model was also fitted, in which the change in each candidate mediator from baseline to week 12 was used to predict the change in inattention symptoms from baseline to week 24. Indirect effects were estimated as the product of the *a* and *b* paths, and 10,000 nonparametric bootstrap resamples were used to derive 95% CIs. These lagged analyses were post hoc exploratory analyses and were not intended to establish causal mediation.

## Results

### Participant Flow and Characteristics

**Figure 1:**
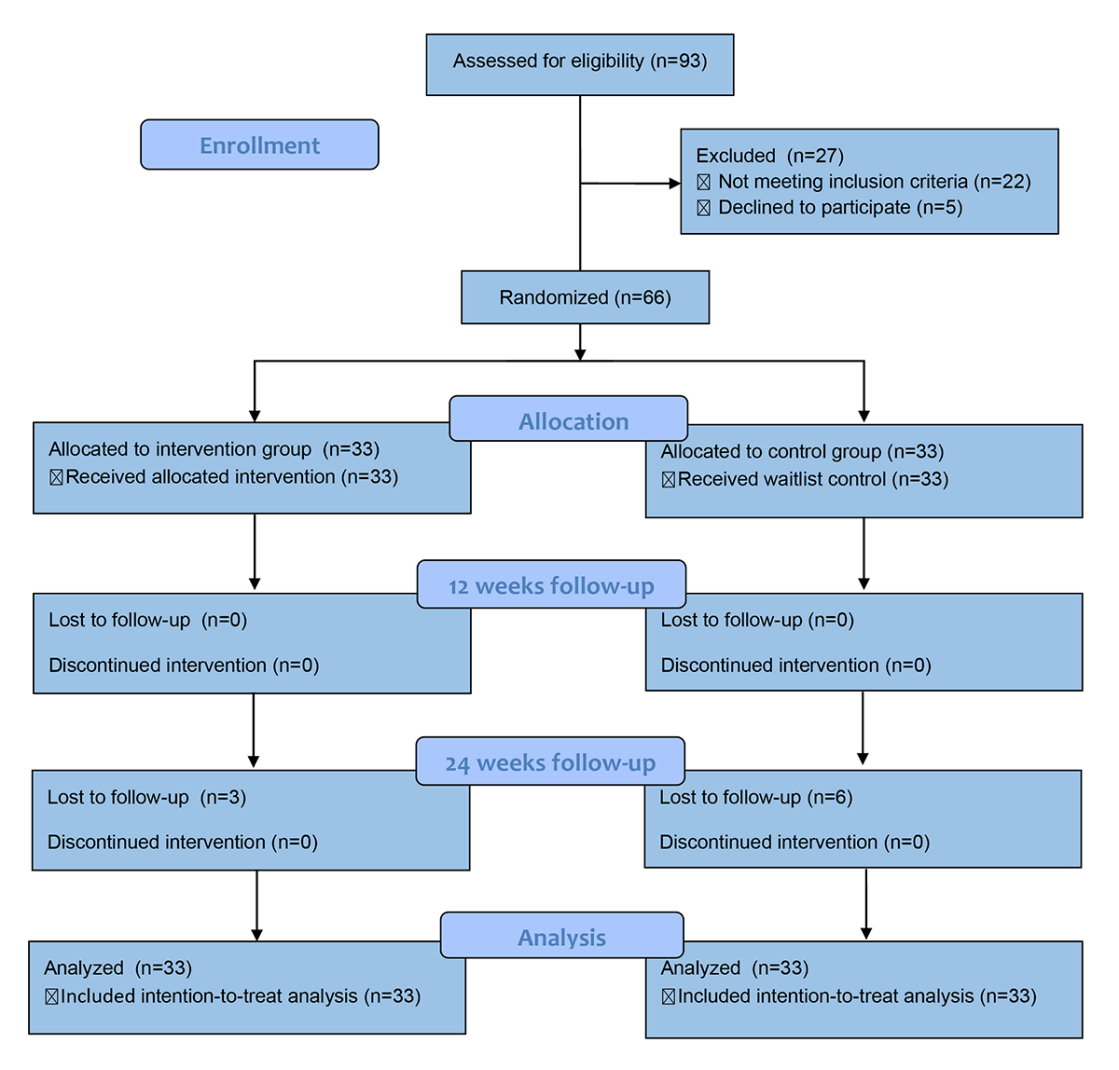
Flow diagram of participants throughout the trial.

A total of 93 children were screened for eligibility (Figure 1). Of these, 27 were excluded: 22 did not meet the inclusion criteria, and 5 declined to participate. The remaining 66 eligible participants were randomly assigned in a 1:1 ratio to the telerehabilitation-based fine motor training group (n=33) or the waitlist control group (n=33). During the intervention and the subsequent 3-month follow-up period, 3 participants in the intervention group and 6 participants in the waitlist control group were lost to follow-up. All randomized participants were included in the intention-to-treat analysis according to their originally assigned groups.

Baseline demographic and clinical characteristics were comparable between the 2 groups (Table 1). No statistically significant between-group differences were observed in age, sex, height, weight, BMI, IQ, medication use, daily MVPA, inattention symptoms, executive function measures, or fine motor skill measures. In the overall sample, the mean age was 8.49 (SD 1.10) years, 77.27% (51/66) of participants were boys, and 12.12% (8/66) were receiving ADHD medication.

**Table 1:** Descriptive characteristics of the participants.

Characteristics			Total (N=66)		Intervention group (n=33)		Control group (n=33)		*P* value	
Age (years), mean (SD)			8.49 (1.10)		8.52 (1.16)		8.46 (1.07)		.83	
**Age group (years), n (%)**										.83
	6-8	40 (60.61)		19 (57.58)		21 (63.64)				
	9-10	26 (39.39)		14 (42.42)		12 (36.36)				
**Sex, n (%)**										.77
	Female	15 (22.73)		8 (24.24)		7 (21.21)				
	Male	51 (77.27)		25 (75.76)		26 (78.79)				
Height (cm), mean (SD)			134.89 (8.99)		135.22 (8.61)		134.55 (9.48)		.76	
Weight (kg), mean (SD)			30.85 (7.78)		31.26 (8.14)		30.45 (7.50)		.68	
BMI (kg/m^2^), mean (SD)			16.77 (3.05)		16.89 (3.25)		16.64 (2.88)		.74	
IQ, mean (SD)			104.05 (11.86)		104.24 (10.40)		103.85 (13.21)		.89	
**Medicine intake, n (%)**										.71
	None	58 (87.88)		30 (90.91)		28 (84.85)				
	Yes	8 (12.12)		3 (9.09)		5 (15.15)				
MVPA^a^ (minutes/day), mean (SD)			74.27 (38.86)		75.33 (38.96)		73.20 (39.35)		.82	
**Core symptom score,** **mean** **(SD)**										
	Inattention	18.77 (3.40)		19.06 (3.79)		18.49 (2.98)		.50		
**Executive functions,** **mean** **(SD)**										
	Inhibitory control	56.01 (13.92)		55.53 (14.19)		56.49 (13.84)		.78		
	Immediate memory	7.89 (4.59)		7.82 (4.99)		7.97 (4.22)		.90		
	Delayed memory	6.65 (4.61)		6.88 (4.36)		6.42 (4.90)		.69		
	Cognitive flexibility	204.14 (49.54)		203.04 (46.22)		205.24 (53.36)		.86		
**Fine motor skills, mean (SD)**										
	Manual dexterity	6.62 (2.52)		6.76 (2.03)		6.48 (2.95)		.66		
	Hand-eye coordination	7.36 (2.32)		7.33 (2.25)		7.39 (2.42)		.92		
	Writing skills	64.53 (11.69)		63.09 (11.16)		65.97 (12.20)		.32		

^a^MVPA: moderate-to-vigorous physical activity.

The intervention comprised 36 scheduled sessions over 12 weeks. Children in the intervention group attended a mean of 30.12 (SD 1.98) sessions, corresponding to 83.67% (30/36) of the scheduled sessions and an average cumulative live training duration of approximately 1800 minutes. The number of attended sessions ranged from 26 to 33. Using the prespecified adherence criterion of attending at least 80% of scheduled sessions, 75.76% (25/33) of children in the intervention group were classified as adherent to the intervention. These children also showed regular homework engagement.

Regarding covariates in the mixed-effects models, age, sex, IQ, and daily MVPA were not statistically significant predictors of any outcome. Medication status was significantly associated only with delayed memory (*F*_1,73.83_=6.22, *P*=.02). All *P* values reported for post hoc pairwise comparisons based on estimated marginal means were adjusted using the false discovery rate method. The *P* values for the main effects of group and time and the group-by-time interaction effects represent omnibus tests from the linear mixed-effects models.

### Effects on Inattention Symptoms

**Figure 2:**
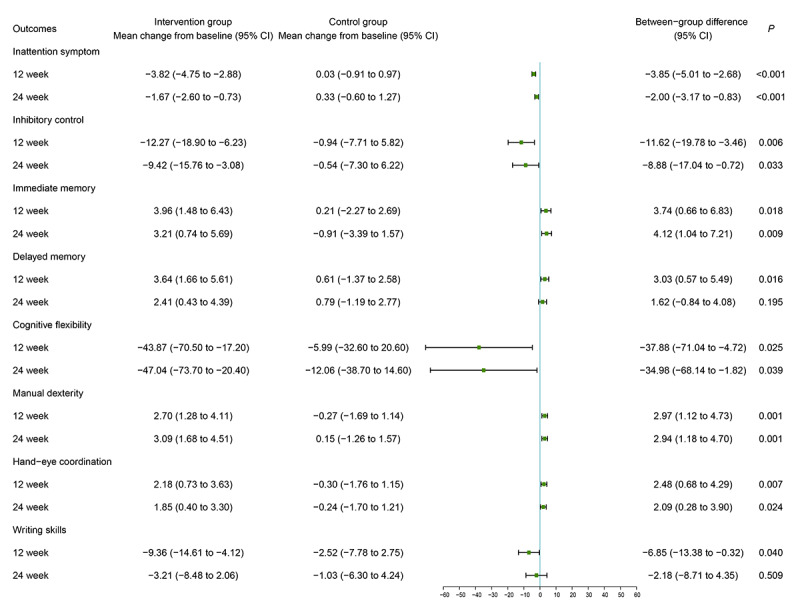
Intervention effects on inattention symptoms, executive functions, and fine motor outcomes at postintervention and 3-month follow-up.

**Figure 3:**
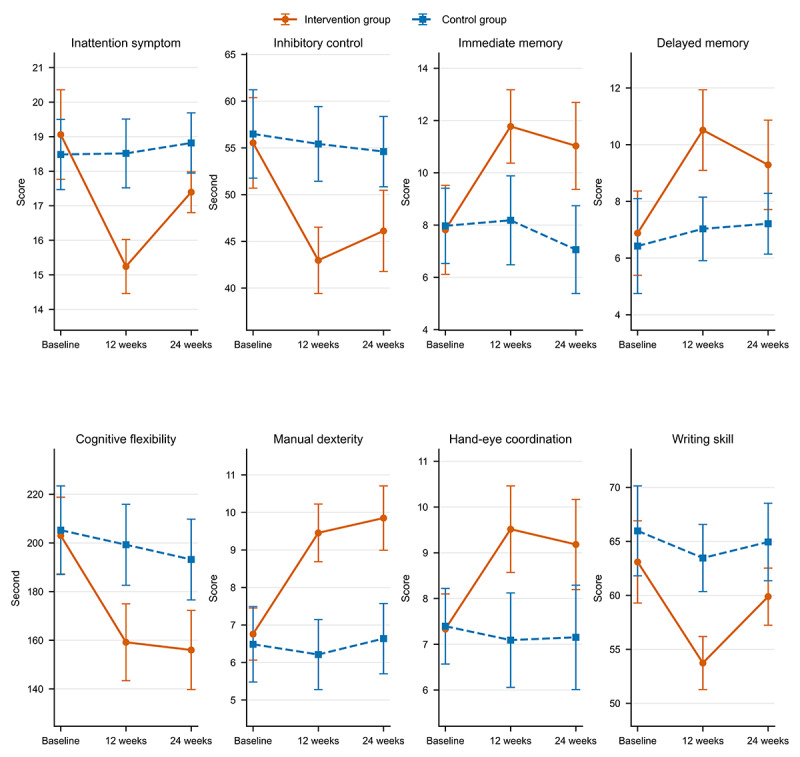
Changes in outcomes over time in the intervention and control groups.

For inattention symptoms, the results showed a significant main effect of group (*F*_1,73.83_=5.75; *P*=.02), a significant main effect of time (*F*_2,136.12_=21.23; *P*<.001), and a significant group-by-time interaction (*F*_2,136.12_=21.30; *P*<.001). In the intervention group, inattention symptoms decreased significantly from baseline at both 12 weeks (MD=−3.82, 95% CI −4.75 to −2.88; *P*<.001) and 24 weeks (MD=−1.67, 95% CI −2.60 to −0.73; *P*=.002) (Figures 2 and 3). In contrast, the control group showed no significant change at either time point. Between-group comparisons showed significantly greater reductions in the intervention group at both 12 weeks (MD=−3.85, 95% CI −5.01 to −2.68; Hedges *g*=−1.13, 95% CI −1.65 to −0.61; *P*<.001) and 24 weeks (MD=−2.00, 95% CI −3.17 to −0.83; Hedges *g*=−0.59, 95% CI −1.08 to −0.10; *P*<.001).

Using the prespecified threshold based on change relative to baseline, the intervention group met the criterion for clinical response at 12 weeks. The mean reduction in the inattention score at 12 weeks was 3.82 points, corresponding to approximately 20% of the baseline value in the intervention group. However, this threshold was no longer met at 24 weeks.

### Effects on Executive Functions

For inhibitory control, the results showed a significant main effect of group (*F*_1,73.83_=11.90; *P*<.001), a significant main effect of time (*F*_2,136.12_=6.68; *P*=.002), and a significant group-by-time interaction (*F*_2,136.12_=4.28; *P*=.02). In the intervention group, significant within-group improvements were observed at both 12 weeks (MD=−12.27, 95% CI −18.90 to −6.23; *P*<.001) and 24 weeks (MD=−9.42, 95% CI −15.76 to −3.08; *P*=.002; Figures 2 and 3). No statistically significant within-group change was observed in the waitlist control group. Between-group comparisons favored the intervention group at both 12 weeks (MD=−11.62, 95% CI −19.78 to −3.46; Hedges *g*=−0.83, 95% CI −1.33 to −0.33; *P*=.006) and 24 weeks (MD=−8.88, 95% CI −17.04 to −0.72; Hedges *g*=−0.63, 95% CI −1.12 to −0.14; *P*=.03).

For immediate memory, the results showed a significant main effect of group (*F*_1,73.83_=12.38; *P*<.001), a significant main effect of time (*F*_2,136.12_=3.59; *P*=.03), and a significant group-by-time interaction (*F*_2,136.12_=4.27; *P*=.02). In the intervention group, significant within-group improvements were observed at both 12 weeks (MD=3.96, 95% CI 1.48-6.43; *P*=.001) and 24 weeks (MD=3.21, 95% CI 0.74-5.69; *P*=.008). No statistically significant within-group change was observed in the waitlist control group. Between-group comparisons favored the intervention group at both 12 weeks (MD=3.74, 95% CI 0.66-6.83; Hedges *g*=0.81, 95% CI 0.31-1.31; *P*=.02) and 24 weeks (MD=4.12, 95% CI 1.04 to 7.21; Hedges *g*=0.89, 95% CI 0.38-1.40; *P*=.009).

For delayed memory, the results showed a significant main effect of group (*F*_1,73.83_=9.08; *P*=.004) and a significant main effect of time (*F*_2,136.12_=6.30; *P*=.002), whereas the group-by-time interaction did not reach statistical significance (*F*_2,136.12_=2.97; *P*=.06). In the intervention group, significant within-group improvements were observed at both 12 weeks (MD=3.64, 95% CI 1.66-5.61; *P*<.001) and 24 weeks (MD=2.41, 95% CI 0.43-4.39; *P*=.01). No statistically significant within-group change was observed in the waitlist control group. The between-group difference was significant at 12 weeks (MD=3.03, 95% CI 0.57-5.49; Hedges *g*=0.65, 95% CI 0.15-1.15; *P*=.02), but not at 24 weeks (MD=1.62, 95% CI −0.84 to 4.08; Hedges *g*=0.35, 95% CI −0.14 to 0.84; *P*=.20).

For cognitive flexibility, the results showed a significant main effect of group (*F*_1,73.83_=13.29; *P*<.001), a significant main effect of time (*F*_2,136.12_=7.19; *P*=.001), and a significant group-by-time interaction (*F*_2,136.12_=3.16; *P*=.04). In the intervention group, significant within-group improvements were observed at both 12 weeks (MD=−43.87, 95% CI −70.50 to −17.20; *P*=.001) and 24 weeks (MD=−47.04, 95% CI −73.70 to −20.40; *P*<.001). No statistically significant within-group change was observed in the waitlist control group. Between-group comparisons favored the intervention group at both 12 weeks (MD=−37.88, 95% CI −71.04 to −4.72; Hedges *g*=−0.76, 95% CI −1.26 to −0.26; *P*=.025) and 24 weeks (MD=−34.98, 95% CI −68.14 to −1.82; Hedges *g*=−0.70, 95% CI −1.20 to −0.20; *P*=.04).

### Effects on Fine Motor Skills

For manual dexterity, the results showed a significant main effect of group (*F*_1,73.83_=37.29; *P*<.001), a significant main effect of time (*F*_2,136.12_=7.27; *P*<.001), and a significant group-by-time interaction (*F*_2,136.12_=7.44; *P*<.001). In the intervention group, significant within-group improvements were observed at both 12 weeks (MD=2.70, 95% CI 1.28-4.11; *P*<.001) and 24 weeks (MD=3.09, 95% CI 1.68-4.51; *P*<.001) (Figures 2 and 3). No statistically significant within-group change was observed in the waitlist control group. Between-group comparisons favored the intervention group at both 12 weeks (MD=2.97, 95% CI 1.12-4.73; Hedges *g*=1.17, 95% CI 0.65-1.69; *P*=.001) and 24 weeks (MD=2.94, 95% CI 1.18-4.70; Hedges *g*=1.16, 95% CI 0.64-1.68; *P*=.001).

For hand-eye coordination, the results showed a significant main effect of group (*F*_1,73.83_=11.66; *P*=.001) and a significant group-by-time interaction (*F*_2,136.12_=4.26; *P*=.02), whereas the main effect of time was not statistically significant (*F*_2,136.12_=2.46; *P*=.09). In the intervention group, significant within-group improvements were observed at both 12 weeks (MD=2.18, 95% CI 0.73-3.63; *P*=0.002) and 24 weeks (MD=1.85, 95% CI 0.40-3.30; *P*=.01). No statistically significant within-group change was observed in the waitlist control group. Between-group comparisons favored the intervention group at both 12 weeks (MD=2.48, 95% CI 0.68-4.29; Hedges *g*=1.06, 95% CI 0.54-1.58; *P*=.007) and 24 weeks (MD=2.09, 95% CI 0.28-3.90; Hedges *g*=0.89, 95% CI 0.38-1.40; *P*=.02).

For writing skills, the results showed a significant main effect of group (*F*_1,73.83_=15.29; *P*<.001) and a significant main effect of time (*F*_2,136.12_=6.65; *P*=.002), whereas the group-by-time interaction was not statistically significant (*F*_2,136.12_=2.25; *P*=.11). In the intervention group, a significant within-group improvement was observed at 12 weeks (MD=−9.36, 95% CI −14.61 to −4.12; *P*<.001), but not at 24 weeks (MD=−3.21, 95% CI −8.48 to 2.06; *P*=.30). The between-group difference favored the intervention group at 12 weeks (MD=−6.85, 95% CI −13.38 to −0.32; Hedges *g*=−0.59, 95% CI −1.08 to −0.10; *P*=.04), but not at 24 weeks (MD=−2.18, 95% CI −8.71 to 4.35; Hedges *g*=−0.19, 95% CI −0.67 to 0.29; *P*=.51).

### Sensitivity Analysis

The sensitivity analysis excluding participants receiving ADHD medication yielded results broadly consistent with the main analysis (Multimedia Appendix 3). Significant between-group differences favoring the intervention group were observed for inattention symptoms at 12 weeks (MD=−4.04, 95% CI −5.31 to −2.76; Hedges *g*=−1.61, 95% CI −2.20 to −1.02; *P*<.001) and 24 weeks (MD=−2.41, 95% CI −3.68 to −1.13; Hedges *g*=−0.96, 95% CI −1.50 to −0.42; *P*<.001), inhibitory control at 12 weeks (MD=−9.07, 95% CI −17.30 to −0.85; Hedges *g*=−0.56, 95% CI −1.09 to −0.03; *P*=.03) and 24 weeks (MD=−8.50, 95% CI −16.73 to −0.28; Hedges *g*=−0.52, 95% CI −1.04 to 0.00; *P*=.04), immediate memory at 12 weeks (MD=3.98, 95% CI 0.50-7.45; Hedges *g*=0.58, 95% CI 0.05-1.11; *P*=.03) and 24 weeks (MD=4.47, 95% CI 0.99-7.95; Hedges *g*=0.65, 95% CI 0.12-1.18; *P*=.01), manual dexterity at 12 weeks (MD=3.38, 95% CI 1.48-5.28; Hedges *g*=0.91, 95% CI 0.37-1.45; *P*=.001) and 24 weeks (MD=3.22, 95% CI 1.32-5.12; Hedges *g*=0.86, 95% CI 0.32-1.40; *P*=.001), and hand-eye coordination at 12 weeks (MD=3.03, 95% CI 1.13-4.92; Hedges *g*=0.81, 95% CI 0.27-1.35; *P*=.002) and 24 weeks (MD=2.59, 95% CI 0.69-4.48; Hedges *g*=0.69, 95% CI 0.16-1.22; *P*=.008). Delayed memory, cognitive flexibility, and writing skill showed significant between-group differences at 12 weeks, but the differences were not statistically significant at 24 weeks.

The adherence-based sensitivity analysis yielded results broadly consistent with the primary intention-to-treat analysis. After excluding participants who attended fewer than 80% of sessions, the analysis included 25 children in the intervention group and 27 children in the control group. The intervention group continued to show greater improvements in inattention symptoms, inhibitory control, immediate memory, cognitive flexibility, manual dexterity, and hand-eye coordination, with effect estimates generally similar in direction and magnitude to those observed in the intention-to-treat analysis. Full results are available in Multimedia Appendix 4.

### Exploratory Age-Based Subgroup Analyses

**Figure 4:**
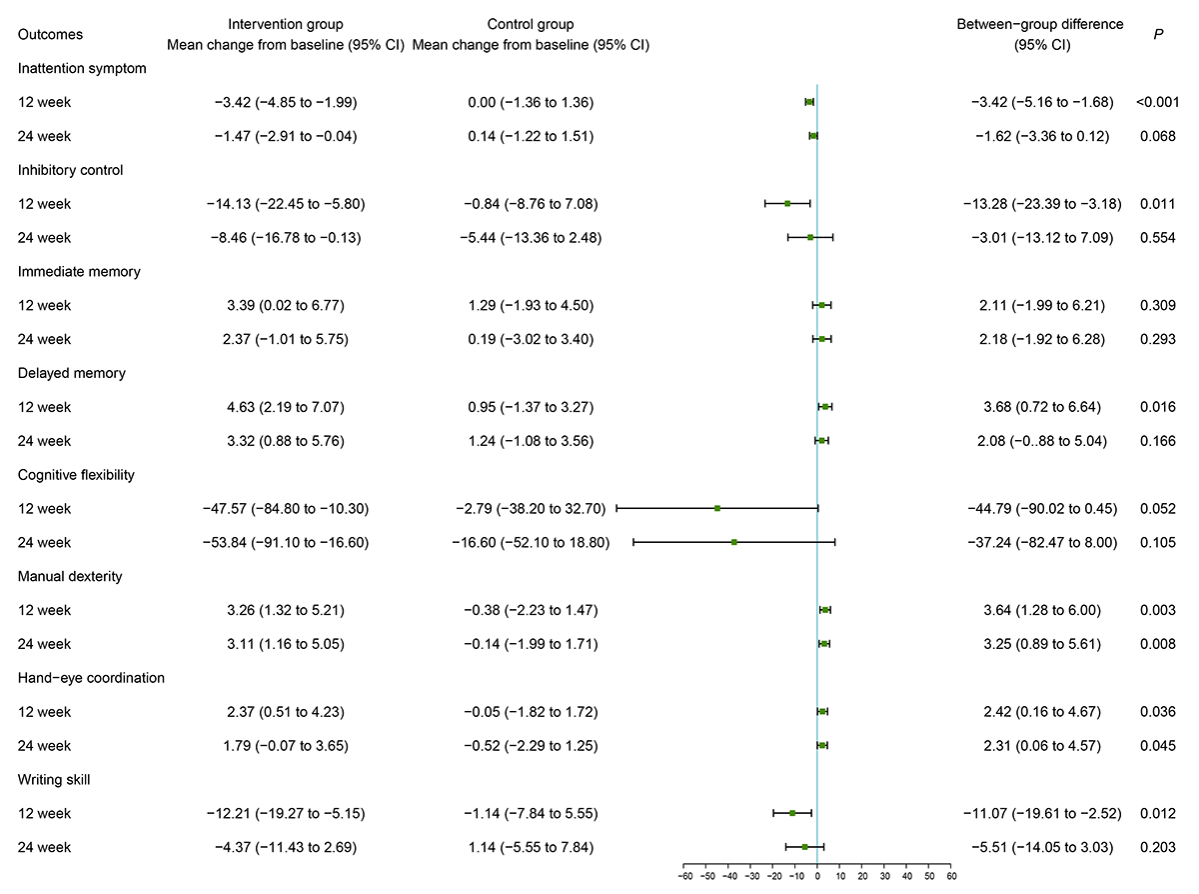
Intervention effects in the subgroup of children aged 6 to 8 years.

In the subgroup of children aged 6 to 8 years (Figure 4), including 19 in the intervention group and 21 in the waitlist control group, compared with the control group, children in the intervention group showed significantly greater improvement in inattention symptoms at 12 weeks (MD=−3.42, 95% CI −5.16 to −1.68; Hedges *g*=−1.20, 95% CI −1.87 to −0.53; *P*<.001), whereas the between-group difference at 24 weeks did not reach statistical significance (MD=−1.62, 95% CI −3.36 to 0.12; Hedges *g*=−0.57, 95% CI −1.20 to 0.06; *P*=.07). Significant between-group differences were observed for inhibitory control at 12 weeks (MD=−13.28, 95% CI −23.39 to −3.18; Hedges *g*=−0.80, 95% CI −1.44 to −0.16; *P*=.01), delayed memory at 12 weeks (MD=3.68, 95% CI 0.72-6.64; Hedges *g*=0.76, 95% CI 0.12-1.40; *P*=.02), manual dexterity at 12 weeks (MD=3.64, 95% CI 1.28-6.00; Hedges *g*=0.94, 95% CI 0.29-1.59; *P*=.003) and 24 weeks (MD=3.25, 95% CI 0.89-5.61; Hedges *g*=0.84, 95% CI 0.19-1.49; *P*=.008), hand-eye coordination at 12 weeks (MD=2.42, 95% CI 0.16-4.67; Hedges *g*=0.65, 95% CI 0.01-1.29; *P*=.04) and 24 weeks (MD=2.31, 95% CI 0.06-4.57; Hedges *g*=0.62, 95% CI 0.02-1.26; *P*=.04), and writing skills at 12 weeks (MD=−11.07, 95% CI −19.61 to −2.52; Hedges *g*=−0.79, 95% CI −1.43 to −0.15; *P*=.01). By contrast, the between-group differences for immediate memory, delayed memory at 24 weeks, cognitive flexibility, and writing skills at 24 weeks were not statistically significant.

**Figure 5:**
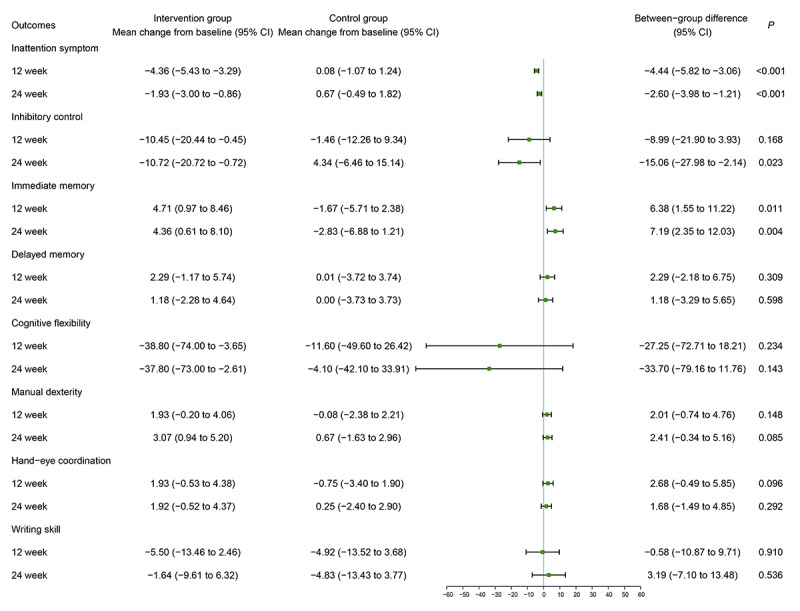
Intervention effects in the subgroup of children aged 9 to 10 years.

In the subgroup of children aged 9 to 10 years (Figure 5), including 14 in the intervention group and 12 in the waitlist control group, compared with the control group, children in the intervention group showed significantly greater reductions in inattention symptoms at both 12 weeks (MD=−4.44, 95% CI −5.82 to −3.06; Hedges *g*=−2.40, 95% CI −3.41 to −1.39; *P*<.001) and 24 weeks (MD=−2.60, 95% CI −3.98 to −1.21; Hedges *g*=−1.40, 95% CI −2.26 to −0.54; *P*<.001). Significant between-group differences were also observed for inhibitory control at 24 weeks (MD=−15.06, 95% CI −27.98 to −2.14; Hedges *g*=−0.87, 95% CI −1.68 to −0.06; *P*=.02), immediate memory at 12 weeks (MD=6.38, 95% CI 1.55-11.22; Hedges *g*=0.99, 95% CI 0.17-1.81; *P*=.011) and 24 weeks (MD=7.19, 95% CI 2.35-12.03; Hedges *g*=1.11, 95% CI 0.28-1.94; *P*=.004). In contrast, no statistically significant between-group differences were found for delayed memory, cognitive flexibility, manual dexterity, hand-eye coordination, or writing skills at either time point.

### Exploratory Correlation Analysis and Mediation Effects

Correlation analyses at week 12 showed that parent-reported inattention symptoms were significantly associated with several executive function domains. Inattention symptoms were positively correlated with inhibitory control impairment (*r*=0.48, 95% CI 0.27-0.65; *P*<.001), indicating that poorer inhibitory control was associated with more severe inattentive symptoms. In contrast, inattention symptoms were negatively correlated with immediate memory (*r*=−0.32, 95% CI −0.52 to −0.09; *P*=.008) and delayed memory (*r*=−0.30, 95% CI −0.51 to −0.07; *P*=.01). No significant correlation was observed between cognitive flexibility and inattention symptoms (*r*=0.03, 95% CI −0.21 to 0.27; *P*=.81).

In the exploratory mediation analyses using week-12 postintervention values, a significant indirect effect was observed through inhibitory control (*β*=−0.85, 95% CI −1.85 to −0.08; *P*=.05), suggesting a statistical indirect association between intervention allocation and postintervention inattention symptoms through inhibitory control. The indirect effects through immediate memory (*β*=−0.32, 95% CI −0.90 to 0.29; *P*=.29), delayed memory (*β*=−0.24, 95% CI −0.87 to 0.39; *P*=.46), and cognitive flexibility (*β*=0.51, 95% CI −0.06 to 1.03; *P*=.07) were not statistically significant. However, the post hoc lagged exploratory mediation analyses did not support longitudinal indirect effects for any candidate mediator. In the lagged level models, the indirect effects were not significant for inhibitory control (*β*=0.13, 95% CI −0.39 to 0.69; *P*=.59), immediate memory (*β*=−0.14, 95% CI −0.54 to 0.30; *P*=.51), delayed memory (*β*=0.01, 95% CI −0.45 to 0.44; *P*>.99), or cognitive flexibility (*β*=0.04, 95% CI −0.38 to 0.42; *P*=.81). The change-score models showed the same pattern. Thus, although the week-12 exploratory model suggested a statistical indirect association through inhibitory control, the lagged analyses did not provide evidence for a longitudinal indirect effect. Detailed results are available in Multimedia Appendix 5.

### Adverse Events Reporting

No adverse events related to the intervention were reported throughout the 12-week training period or during follow-up.

## Discussion

### Overview

In this trial, a 12-week telerehabilitation-based fine motor training program was associated with greater improvements in parent-reported inattention symptoms, selected executive function outcomes, and fine motor skills in children aged 6 to 10 years with inattentive ADHD compared with a waitlist control condition. The reduction in inattention symptoms was strongest immediately after the intervention and remained statistically significant, although attenuated, at the 3-month follow-up. Improvements in executive functions were observed for inhibitory control, immediate memory, and cognitive flexibility at both postintervention assessments, whereas delayed memory improved only immediately after the intervention. For fine motor outcomes, the most consistent improvements were found in manual dexterity and hand-eye coordination, while writing skills improved only immediately after the intervention. Sensitivity analysis excluding children receiving ADHD medication showed a broadly similar pattern, supporting the robustness of the main findings. The standardized effect sizes provided additional context beyond statistical significance, with large effects observed for manual dexterity and hand-eye coordination, consistent with the intervention’s direct focus on fine motor and visuomotor practice. The effect on parent-reported inattention symptoms was large at 12 weeks and moderate at 24 weeks; however, the prespecified 20% clinical response threshold was met only at 12 weeks. Therefore, although the findings suggest potentially meaningful short-term improvements, their clinical relevance should be interpreted cautiously. In particular, because the intervention was compared with a waitlist control condition and involved a multicomponent package of supervised online sessions, therapist feedback, structured practice, parental support, and homework assignments, the observed improvements cannot be attributed solely to fine motor training. Repeated therapist contact may have enhanced children’s motivation, task persistence, confidence, and adherence through real-time feedback, encouragement, and individualized task adjustment. Family engagement may also have increased routine structure, practice opportunities, homework completion, and parental awareness of children’s behavior and progress. These nonspecific factors, together with social interaction and treatment expectancy, may have contributed to changes in parent-reported inattention symptoms and fine motor outcomes independent of the specific fine motor training content. Accordingly, the findings should be viewed as evidence of the potential benefit of a telerehabilitation-based fine motor intervention package rather than definitive evidence of the specific efficacy of fine motor training alone.

The reduction in inattention symptoms is clinically relevant because inattentive symptoms are closely related to task persistence, response monitoring, and everyday functional difficulties in children with inattentive ADHD. In this study, the intervention group showed a statistically significant reduction in parent-reported inattention symptoms, and the mean postintervention change reached the prespecified threshold for clinical response. This finding is broadly consistent with previous evidence suggesting that movement-based and cognitively engaging interventions may improve ADHD-related symptoms in children [7,17,33,34]. However, the effect was attenuated at the 3-month follow-up, suggesting that continued practice, booster sessions, or the integration of training activities into daily routines may be needed to sustain symptom-related gains.

The intervention was also associated with improvements in several executive function domains. This may be related to the cognitive demands embedded in the fine motor tasks. Although fine motor training is often considered a motor-based intervention, many tasks require children to maintain attention, follow multistep instructions, inhibit premature responses, monitor accuracy, regulate speed and force, and adjust movements according to visual feedback [35]. These processes overlap with inhibitory control, working memory-related visual memory, and cognitive flexibility [23,35]. Therefore, the present findings suggest that structured fine motor activities with embedded cognitive demands may be associated with improvements in selected executive function outcomes. Importantly, the present intervention should be distinguished from conventional aerobic or moderate-to-vigorous exercise programs. Its rationale was not based primarily on physical intensity, aerobic fitness, or exercise dose. Instead, the program emphasized fine motor control, visuomotor integration, sequencing, inhibition, and performance monitoring. Therefore, the findings should not be interpreted as evidence that low-intensity physical exercise improves executive functions. Rather, they suggest that rehabilitation-oriented fine motor practice may provide a cognitively meaningful training context for children with inattentive ADHD.

The intervention produced the most consistent benefits in manual dexterity and hand-eye coordination. These findings are expected because the training directly targeted precise hand manipulation, visuomotor integration, bimanual coordination, and speed-accuracy control. These abilities are relevant to many school- and home-based activities, such as handwriting, tool use, object manipulation, dressing, eating with utensils, play, and classroom tasks. From an occupational performance perspective, improvements in manual dexterity and hand-eye coordination may therefore have potential functional relevance beyond isolated motor test performance [18,19]. However, because occupational performance, participation, school functioning, activities of daily living, and quality of life were not directly assessed in this trial, such functional implications should be interpreted cautiously. In contrast, the improvement in writing skills was observed immediately after the intervention but was not sustained at the 3-month follow-up. Writing is a complex functional skill that depends not only on fine motor control but also on visuomotor integration, attention, planning, orthographic knowledge, writing fluency, and repeated practice in meaningful academic contexts [18]. A 12-week fine motor program may improve underlying graphomotor capacities, but this may not be sufficient to produce sustained improvement in functional handwriting performance without continued practice or school-based transfer activities. Future interventions may need to include more explicit handwriting practice, teacher involvement, classroom-based transfer tasks, or booster sessions to promote durable gains in writing-related outcomes. Future trials should also incorporate occupation-centered and ecologically valid outcome measures to determine whether improvements in fine motor outcomes translate into meaningful functional gains.

The exploratory mediation analysis suggested a statistical indirect association through inhibitory control at the 12-week postintervention assessment. This finding is theoretically plausible because fine motor training tasks require children to inhibit premature responses, follow task rules, regulate movement speed, and prioritize accuracy, all of which are closely related to inhibitory control [8,36]. However, this finding should be interpreted with substantial caution. In the original mediation model, inhibitory control and inattention symptoms were measured at the same postintervention time point; therefore, the temporal sequence between the candidate mediator and outcome could not be established. Reverse or bidirectional explanations remain possible. For example, improvements in inattentive symptoms may have facilitated better inhibitory control performance, or both improvements may have reflected broader nonspecific effects of structured practice, therapist contact, parental involvement, or treatment expectancy. Consistent with this caution, the post hoc lagged exploratory mediation analyses did not support longitudinal indirect effects through inhibitory control, immediate memory, delayed memory, or cognitive flexibility. Therefore, the mediation findings should be regarded as exploratory and hypothesis-generating rather than as evidence of a causal mechanism. Future trials should include larger samples and repeated, temporally separated assessments of both candidate mediators and outcomes to formally test longitudinal mediation pathways.

The exploratory age-based subgroup analyses suggested possible differences in the pattern of intervention-related changes between younger and older children. However, these findings should be interpreted with substantial caution. The subgroup of children aged 6 to 8 years included 19 children in the intervention group and 21 in the control group, whereas the subgroup of children aged 9 to 10 years included only 14 and 12 children, respectively. These small subgroup sizes increase the risk of unstable effect estimates and reduce the precision of between-group comparisons, as reflected by the relatively wide CIs. Moreover, the trial was not powered to test age-by-treatment interaction effects. Therefore, the subgroup findings should be viewed as exploratory and hypothesis-generating rather than as evidence of age-specific treatment effects. Larger trials with prespecified moderator analyses and sufficient statistical power are needed to determine whether age or developmental stage influences response to telerehabilitation-based fine motor training.

The telerehabilitation format may support real-world implementation by reducing travel burden, improving scheduling flexibility, and allowing children to practice fine motor activities in the home environment using simple materials. This model may be particularly useful for families with limited access to center-based pediatric rehabilitation services. Live online delivery also allows therapists to provide real-time feedback, monitor performance, and adjust task difficulty while involving caregivers in home-based practice. Nevertheless, broader implementation would require attention to several practical issues. These include therapist training, intervention fidelity, stable internet access, caregiver availability, privacy protection, adherence monitoring, and integration with existing clinical or school-based services. The program may be most appropriate as an adjunctive intervention rather than a replacement for individualized rehabilitation or educational support. Future pragmatic trials should evaluate scalability, cost-effectiveness, therapist workload, family acceptability, digital accessibility, and whether school-based transfer tasks or booster sessions can enhance long-term functional benefits.

Several limitations should be acknowledged. First, the sample size was modest, and the study was not powered for subgroup or mediation analyses. The sample size calculation was based on effect size estimates from broader exercise intervention studies rather than directly comparable fine motor telerehabilitation trials, which may limit the precision of the sample size justification. Second, the study used a waitlist control group rather than an active control condition. Therefore, nonspecific effects, such as therapist contact, parental attention, structured routines, family engagement, social interaction, and treatment expectancy, could not be fully separated from the specific effects of fine motor training. Future trials should include active comparator conditions matched for contact time, structure, and family involvement. Third, inattention symptoms were assessed using parent-reported SNAP-IV scores only. Although parents were well positioned to observe children’s behavior in the home environment where the intervention was delivered, parent ratings may have been influenced by expectancy effects and may not fully capture functioning in school settings. Teacher-reported outcomes were not collected because participants were recruited outside the school setting, and objective attention measures were considered during study planning but were not included because of feasibility concerns regarding standardized administration, remote testing conditions, and assessment burden. Future trials should include teacher-rated ADHD symptoms, objective attention measures, and classroom-based behavioral observations. Fourth, although several procedures were used to support intervention fidelity, including a standardized manual, instructor training, attendance logs, real-time correction, and homework documentation, formal independent fidelity ratings were not collected. Future studies should incorporate structured fidelity checklists, independent ratings of recorded sessions, and quantitative reporting of therapist adherence and delivery quality. Fifth, the intervention was delivered through a single digital platform, Tencent Meeting. Although this platform was accessible to participating families and supported real-time interaction, the findings may not generalize to other telerehabilitation platforms with different functions, usability, privacy settings, connection stability, or monitoring capabilities. Finally, the follow-up period was limited to 3 months, and longer-term maintenance remains uncertain.

Despite these limitations, this study has several implications. Conceptually, it supports the view that fine motor training may serve not only as a motor intervention but also as a structured, cognitively engaging approach for children with inattentive ADHD. Clinically, the findings suggest that telerehabilitation-based fine motor training may be a feasible and potentially beneficial adjunctive approach that can be delivered outside traditional rehabilitation settings. This may improve accessibility for children and families who face barriers to in-person services.

### Conclusion

In conclusion, this RCT provides preliminary evidence that a 12-week telerehabilitation-based fine motor training program may be a feasible and potentially beneficial adjunctive intervention for children aged 6 to 10 years with inattentive ADHD. Compared with a waitlist control condition, the intervention was associated with greater improvements in parent-reported inattention symptoms, selected executive function outcomes, and fine motor skills. However, these findings should be interpreted cautiously because of the waitlist control design, reliance on parent-reported symptom outcomes, and the exploratory nature of the mediation and subgroup analyses. The observed benefits should therefore be considered preliminary until confirmed in larger trials with active comparator groups matched for therapist contact, family engagement, structured activity exposure, and treatment expectancy. Future studies should also incorporate objective attention measures, teacher-reported outcomes, participation-based measures, rigorous fidelity assessment, and longer-term follow-up to clarify the clinical relevance, specific active ingredients, and scalability of this intervention.

## Data Availability

The datasets generated or analyzed during this study are available from the corresponding author on reasonable request.

## Appendix

Multimedia Appendix 1 CONSORT-eHEALTH checklist (V 1.6.1).

Multimedia Appendix 2 Executive function assessment materials used in the study.

Multimedia Appendix 3 Intervention effects in the sensitivity analysis excluding participants receiving medication.

Multimedia Appendix 4 Adherence-based sensitivity analysis excluding participants who attended fewer than 80% of sessions.

Multimedia Appendix 5 Exploratory mediation analyses in the intention-to-treat sample.

## Abbreviations

ADHD: attention-deficit/hyperactivity disorder
CONSORT: Consolidated Standards of Reporting Trials
CONSORT-EHEALTH: Consolidated Standards of Reporting Trials of Electronic and Mobile Health Applications and Online Telehealth
MD: mean difference
MVPA: moderate-to-vigorous physical activity
RCT: randomized controlled trial
SNAP-IV: Swanson, Nolan, and Pelham IV Rating Scale
THPC: Tseng Handwriting Problem Checklist
