# Supplementary material for: A Telerehabilitation-Based Fine Motor Training Program for Children With Inattentive Attention-Deficit/Hyperactivity Disorder: Randomized Controlled Trial
Source: J Med Internet Res. 2026 Jul 23;28:e97365. doi: 10.2196/97365 (PMC13448201; doi:10.2196/97365)
Supplement: Supplementary file 2 [file jmir_v28i1e97365_app2.pdf]

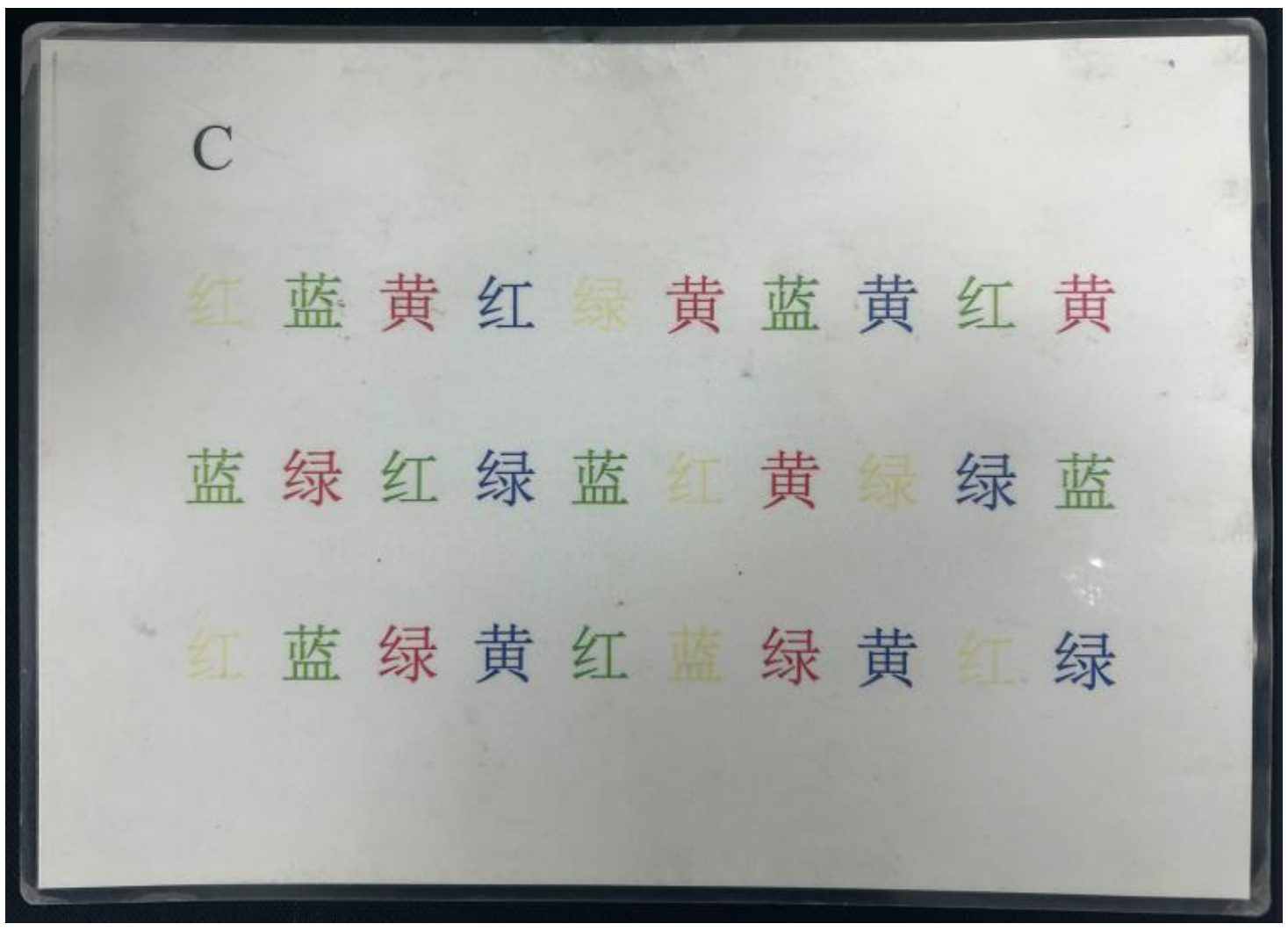

Supplementary Material Fig 1. Stroop Color and Word Test materials

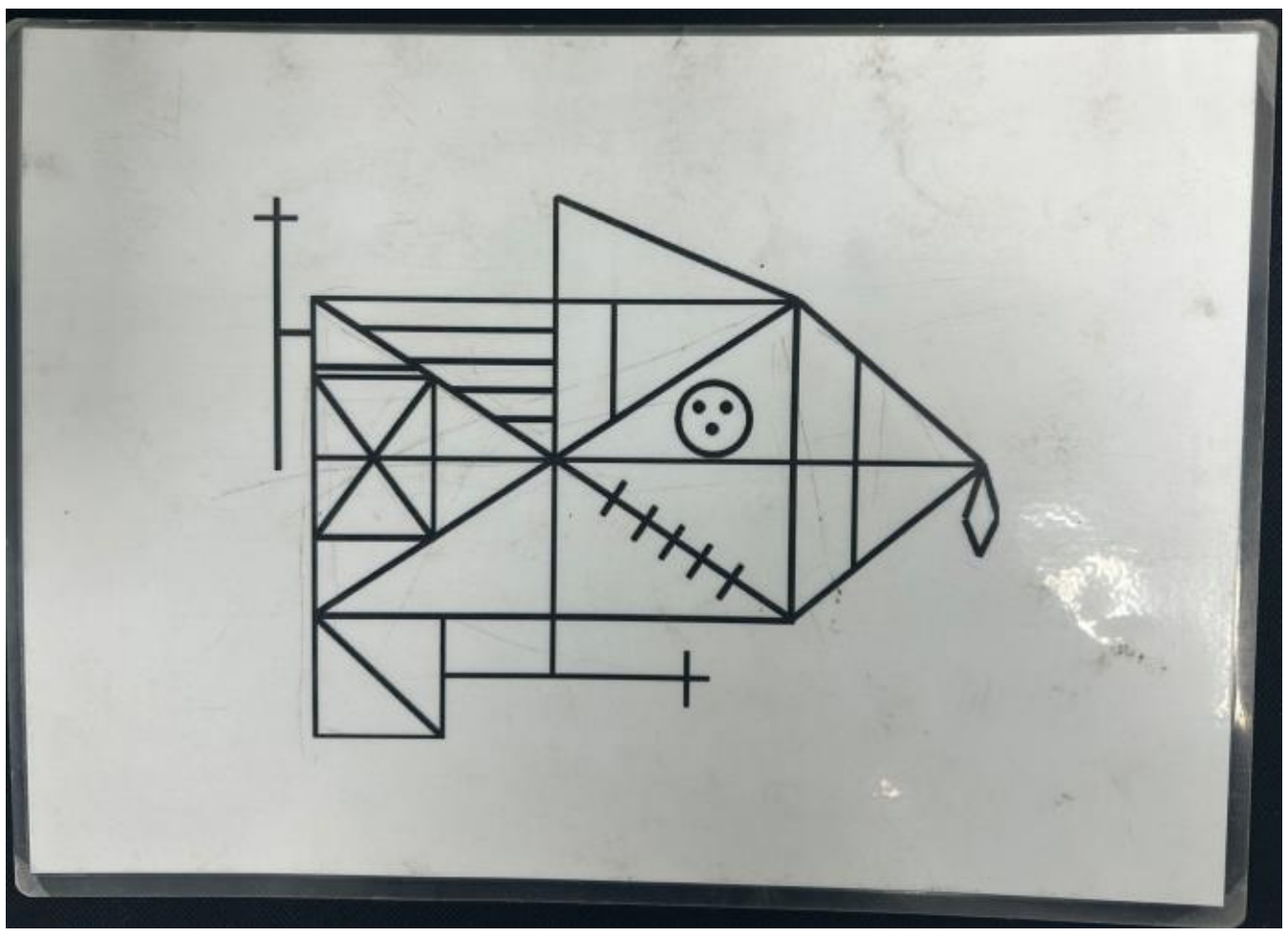

Supplementary Material Fig 2. Rey-Osterrieth Complex Figure Test materials

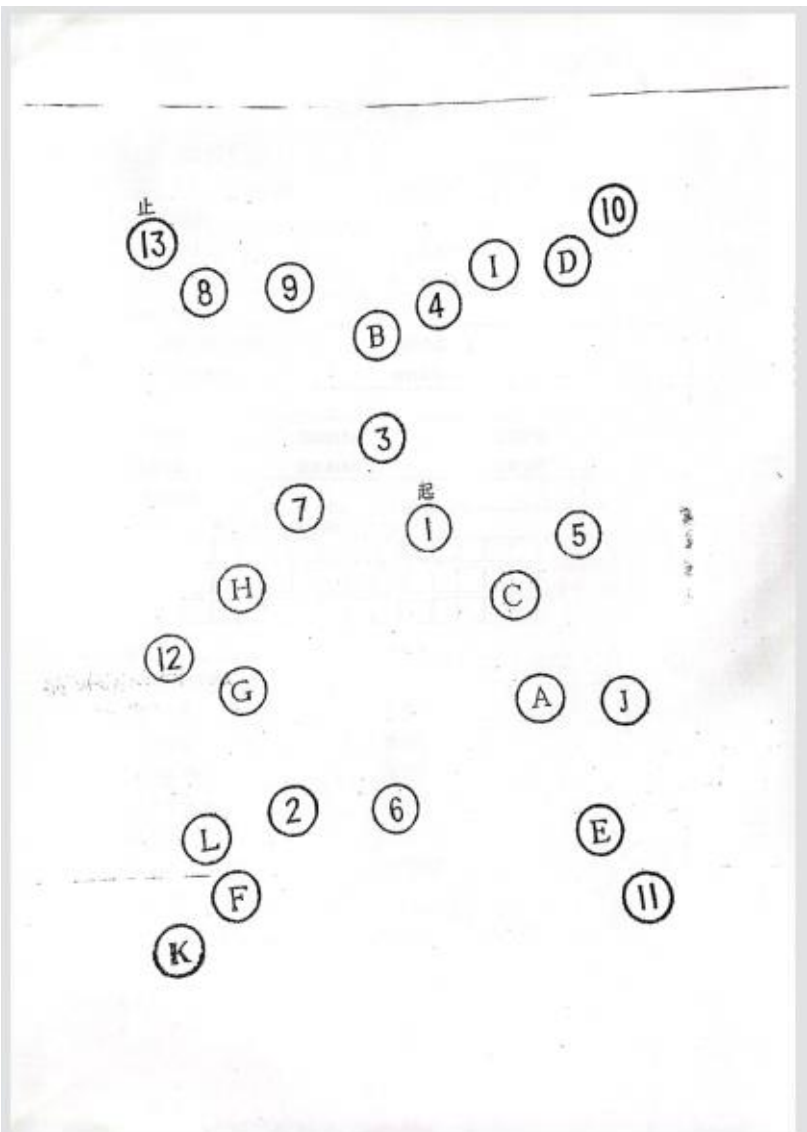

Supplementary Material Fig 3. Trail Making Test materials
