# Supplementary material for: A Telerehabilitation-Based Fine Motor Training Program for Children With Inattentive Attention-Deficit/Hyperactivity Disorder: Randomized Controlled Trial
Source: J Med Internet Res. 2026 Jul 23;28:e97365. doi: 10.2196/97365 (PMC13448201; doi:10.2196/97365)
Supplement: Supplementary file 3 [file jmir_v28i1e97365_app3.pdf]

Outcomes	Intervention group Mean change from baseline (95% CI)	Control group Mean change from baseline (95% CI)	Between−group difference (95% CI)	*P*
Inattention symptom				
12 week	−4.00 (−5.01 to −2.99)	0.04 (−1.01 to 1.08)	−4.04 (−5.31 to −2.76)	<0.001
24 week	−1.77 (−2.77 to −0.76)	0.64 (−0.40 to 1.68)	−2.41 (−3.68 to −1.13)	<0.001
Inhibitory control				
12 week	−12.09 (−18.59 to −5.60)	−3.02 (−9.74 to 3.70)	−9.07 (−17.30 to −0.85)	0.031
24 week	−11.56 (−18.05 to −5.07)	−3.06 (−9.78 to 3.66)	−8.50 (−16.73 to −0.28)	0.042
Immediate memory				
12 week	4.08 (1.34 to 6.83)	0.11 (−2.73 to 2.95)	3.98 (0.50 to 7.45)	0.025
24 week	3.40 (0.66 to 6.14)	−1.07 (−3.91 to 1.77)	4.47 (0.99 to 7.95)	0.012
Delayed memory				
12 week	4.00 (1.85 to 6.15)	0.50 (−1.72 to 2.72)	3.50 (0.78 to 6.22)	0.012
24 week	2.48 (0.34 to 4.63)	1.07 (−1.15 to 3.29)	1.41 (−1.31 to 4.13)	0.306
Cognitive flexibility				
12 week	−50.28 (−78.30 to −22.20)	2.87 (−26.10 to 31.90)	−53.15 (−88.66 to −17.64)	0.004
24 week	−50.08 (−78.10 to −22.00)	−14.97 (−44.00 to 14.00)	−35.11 (−70.62 to 0.40)	0.053
Manual dexterity				
12 week	2.67 (1.17 to 4.17)	−0.71 (−2.27 to 0.84)	3.38 (1.48 to 5.28)	0.001
24 week	2.90 (1.40 to 4.40)	−0.32 (−1.88 to 1.23)	3.22 (1.32 to 5.12)	0.001
Hand−eye coordination				
12 week	2.10 (0.61 to 3.60)	−0.93 (−2.48 to 0.62)	3.03 (1.13 to 4.92)	0.002
24 week	1.77 (0.27 to 3.26)	−0.82 (−2.37 to 0.73)	2.59 (0.69 to 4.48)	0.008
Writing skill				
12 week	−9.71 (−15.13 to −4.29)	−1.93 (−7.74 to 3.89)	−7.78 (−14.78 to −0.79)	0.030
24 week	−3.10 (−8.52 to 2.33)	−0.26 (−6.07 to 5.55)	−2.84 (−9.84 to 4.16)	0.423

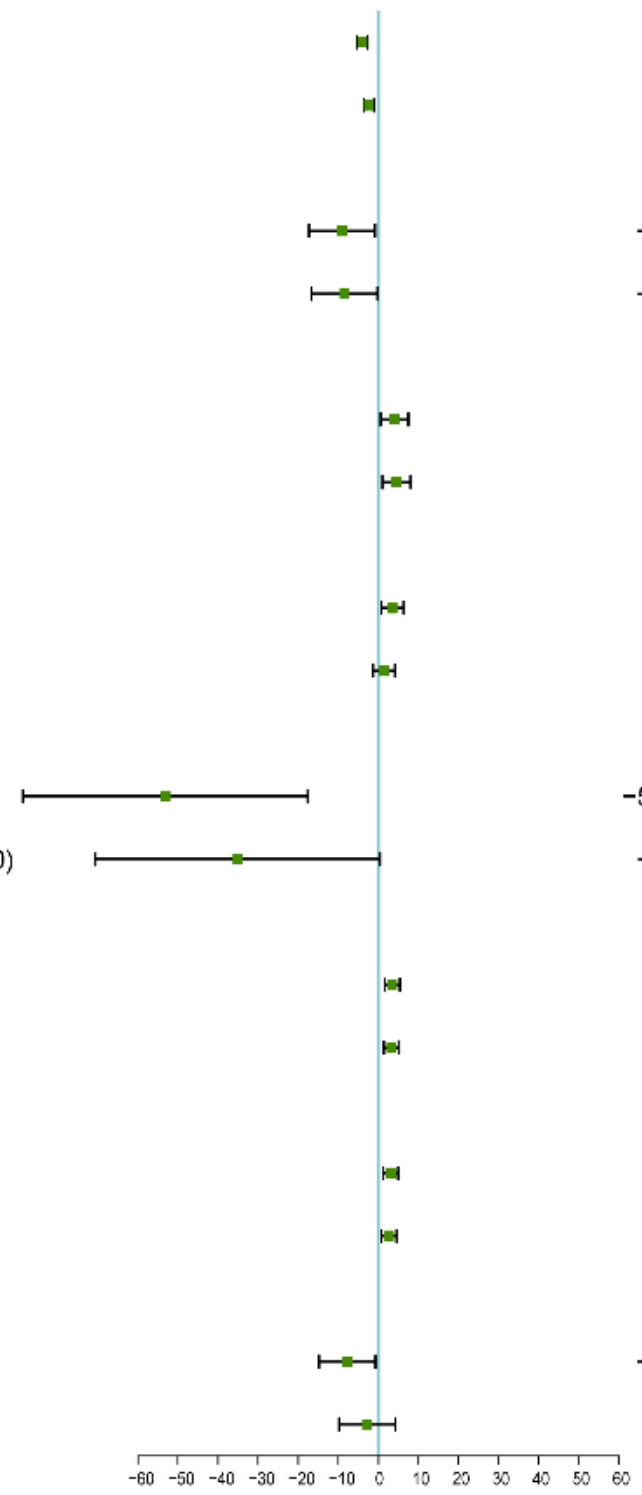

Supplementary Material Fig 1. Intervention effects in the sensitivity analysis excluding participants receiving medication
