# Supplementary material for: A Telerehabilitation-Based Fine Motor Training Program for Children With Inattentive Attention-Deficit/Hyperactivity Disorder: Randomized Controlled Trial
Source: J Med Internet Res. 2026 Jul 23;28:e97365. doi: 10.2196/97365 (PMC13448201; doi:10.2196/97365)
Supplement: Supplementary file 4 [file jmir_v28i1e97365_app4.pdf]

Supplementary Material Table 1. Adherence-based sensitivity analysis excluding participants who attended fewer than 80% of sessions

Outcome	Time point	Intervention, n	Control, n	Intervention change	Control change	MD (95% CI)	Hedges' g	P value
Inattention symptom	Week 12	25	27	-3.68 (2.56)	-0.15 (1.26)	-3.53 (-4.68 to -2.38)	-1.02 (-1.42 to -0.72)	<0.001
Inattention symptom	Week 24	25	27	-2.28 (2.88)	-0.15 (2.33)	-2.13 (-3.60 to -0.66)	-0.61 (-1.07 to -0.22)	0.005
Inhibitory control	Week 12	25	27	-11.66 (15.10)	0.52 (15.56)	-12.18 (-20.72 to -3.63)	-0.89 (-1.59 to -0.30)	0.006
Inhibitory control	Week 24	25	27	-12.33 (14.58)	0.12 (14.09)	-12.45 (-20.45 to -4.45)	-0.91 (-1.60 to -0.36)	0.003
Immediate memory	Week 12	25	27	5.92 (5.87)	0.93 (6.55)	4.99 (1.53 to 8.46)	1.02 (0.35 to 1.86)	0.006
Immediate memory	Week 24	25	27	4.24 (5.90)	-0.93 (6.91)	5.17 (1.59 to 8.74)	1.06 (0.38 to 1.89)	0.005
Delayed memory	Week 12	25	27	3.52 (5.80)	0.81 (5.38)	2.71 (0.22 to 5.63)	0.58 (0.05 to 1.24)	0.048
Delayed memory	Week 24	25	27	2.66 (4.95)	1.19 (5.30)	1.47 (-1.38 to 4.33)	0.32 (-0.28 to 0.92)	0.304
Cognitive flexibility	Week 12	25	27	-55.80 (44.14)	-6.98 (81.84)	-48.82 (-85.29 to -12.34)	-0.96 (-1.83 to -0.28)	0.010
Cognitive flexibility	Week 24	25	27	-58.37 (50.07)	-19.61 (77.99)	-38.76 (-75.10 to -2.41)	-0.76 (-1.60 to -0.08)	0.037
Manual dexterity	Week 12	25	27	3.08 (2.83)	-0.22 (4.89)	3.30 (1.09 to 5.52)	1.25 (0.41 to 2.40)	0.004
Manual dexterity	Week 24	25	27	3.96 (2.82)	0.11 (4.59)	3.85 (1.73 to 5.96)	1.45 (0.67 to 2.54)	0.001
Hand-eye coordination	Week 12	25	27	2.52 (2.84)	-0.30 (4.03)	2.82 (0.88 to 4.75)	1.20 (0.43 to 2.05)	0.005
Hand-eye coordination	Week 24	25	27	2.44 (3.33)	-0.41 (3.51)	2.85 (0.94 to 4.75)	1.22 (0.43 to 2.18)	0.004
Writing skill	Week 12	25	27	-11.92 (8.64)	-4.15 (14.88)	-7.77 (-14.52 to -1.03)	-0.68 (-1.28 to -0.12)	0.025
Writing skill	Week 24	25	27	-4.36 (9.72)	-1.70 (17.42)	-2.66 (-10.48 to 5.17)	-0.23 (-0.88 to 0.46)	0.497

Note. Values are mean (SD) unless otherwise indicated.
