# Supplementary material for: A Telerehabilitation-Based Fine Motor Training Program for Children With Inattentive Attention-Deficit/Hyperactivity Disorder: Randomized Controlled Trial
Source: J Med Internet Res. 2026 Jul 23;28:e97365. doi: 10.2196/97365 (PMC13448201; doi:10.2196/97365)
Supplement: Supplementary file 5 [file jmir_v28i1e97365_app5.pdf]

Supplementary Material Table 1. Results of the simple mediation models (Fine motor training → Executive functions → SNAP-IV inattention scores)

Mediation model	Estimate (β), 95% CI			Correlation analysis between executive functions and inattention	
	Total effect	Direct effect	Indirect effect	r	P
IV→ IC →Inattention	-3.27 (-4.52 to -2.04)	-2.42 (-3.75 to -1.03)	-0.85 (-1.85 to -0.08)	0.48 (0.27 to 0.65)	<0.001
IV→ IM →Inattention	-3.27 (-4.49 to -2.04)	-2.95 (-4.25 to -1.79)	-0.32 (-0.90 to 0.29)	-0.32 (-0.52 to -0.09)	0.008
IV→ DM →Inattention	-3.27 (-4.52 to -2.04)	-3.03 (-4.43 to -1.70)	-0.24 (-0.87 to 0.39)	-0.30 (-0.51 to -0.07)	0.013
IV→ CF →Inattention	-3.27 (-4.53 to -1.99)	-3.79 (-5.05 to -2.31)	0.51 (-0.06 to 1.03)	0.03 (-0.21 to 0.27)	0.811

IV independent variable, *IC* inhibitory control, *IM* immediate memory, *DM* delayed memory, *CF* cognitive flexibility

Supplementary Material Table 2. Post hoc lagged exploratory mediation analyses for all candidate mediators in the intention-to-treat sample

Mediator	Model	a path, β (95% CI)	a path P	b path, β (95% CI)	b path P	Direct effect, β (95% CI)	Direct effect P	Indirect effect, β	Bootstrap 95% CI	Bootstrap P
IC	Lagged level model	-12.30 (-17.68 to -6.92)	<0.001	-0.01 (-0.06 to 0.04)	0.641	-1.80 (-2.88 to -0.71)	0.002	0.13	-0.39 to 0.69	0.593
IC	Change-score model	-12.30 (-17.68 to -6.92)	<0.001	0.00 (-0.03 to 0.03)	0.823	-1.60 (-2.55 to -0.64)	0.001	-0.04	-0.37 to 0.27	0.776
IM	Lagged level model	3.59 (1.32 to 5.85)	0.002	-0.04 (-0.14 to 0.06)	0.435	-1.50 (-2.46 to -0.54)	0.003	-0.14	-0.54 to 0.30	0.513
IM	Change-score model	3.59 (1.32 to 5.85)	0.002	-0.01 (-0.08 to 0.06)	0.786	-1.60 (-2.53 to -0.67)	0.001	-0.03	-0.26 to 0.26	0.825
DM	Lagged level model	3.41 (1.59 to 5.24)	<0.001	0.00 (-0.12 to 0.13)	0.969	-1.65 (-2.65 to -0.65)	0.002	0.01	-0.45 to 0.44	0.999
DM	Change-score model	3.41 (1.59 to 5.24)	<0.001	-0.01 (-0.10 to 0.07)	0.771	-1.60 (-2.53 to -0.66)	0.001	-0.04	-0.48 to 0.28	0.775
CF	Lagged level model	-40.29 (-63.76 to -16.83)	0.001	-0.00 (-0.01 to 0.01)	0.822	-1.68 (-2.66 to -0.71)	0.001	0.04	-0.38 to 0.42	0.814
CF	Change-score model	-40.29 (-63.76 to -16.83)	0.001	0.00 (-0.01 to 0.01)	0.949	-1.63 (-2.55 to -0.71)	<0.001	-0.01	-0.33 to 0.27	0.949

Note. The intervention group was coded as 1 and the wait-list control group as 0. IC=inhibitory control; IM=immediate memory; DM=delayed memory; CF=cognitive flexibility. In the lagged level model, each week-12 executive function variable was entered as the candidate mediator and week-24 inattention symptoms as the outcome, with adjustment for the corresponding baseline mediator value and baseline inattention symptoms. In the change-score model, change in each candidate mediator from baseline to week 12 was entered as the mediator and change in inattention symptoms from baseline to week 24 as the outcome. Indirect effects were estimated as the product of the a and b paths using 10,000 nonparametric bootstrap resamples. These analyses were post hoc, exploratory, and should not be interpreted as establishing causal mediation. Overall interpretation: none of the candidate executive function mediators showed evidence of a longitudinal indirect effect because all bootstrap 95% CIs for the indirect effects included 0.
